# Sexual Dimorphism and Sex-2D : 4D Interactions on Fasting Lipid Variables in an Adult Ghanaian Population

**DOI:** 10.1155/2022/3303588

**Published:** 2022-06-23

**Authors:** Moses Banyeh, Nafiu Amidu, Draman Abdul-Wahab, Abdul-Salim Zakaria

**Affiliations:** Department of Biomedical Laboratory Science, University for Development Studies, Tamale, Ghana

## Abstract

Prenatal hormone exposure has been suggested as a correlate of adult circulating estrogen and testosterone. If this observation is true, then prenatal hormone exposure may have an association with lipid homeostasis in adulthood. The study sought to investigate sexual dimorphism and the interactions between the putative marker of prenatal hormone exposure (2D : 4D) and sex on adult fasting plasma lipid variables. The study was cross-sectional from June to December 2021 at the University for Development Studies. The participants were between 18 and 30 years of age and consisted of 206 healthy persons (female = 93, male = 113). The right hand (2D : 4DR), the left hand (2D : 4DL), and the right-left 2D : 4D difference (Dr-l) were measured using computer-assisted analysis. Fasting venous blood samples were collected and analyzed for lipid variables including total cholesterol (TCHOL) and high-density lipoprotein cholesterol (HDL-C). There were no significant differences in the 2D : 4D ratio and lipid variables between males and females. However, after adjusting for age and BMI, the 2D : 4DR (*P* = 0.014) and the 2D : 4DL (*P* = 0.007) increased with increasing fasting plasma HDL-C on average. Moreover, there were significant interactions between sex and the 2D : 4DR (*P* = 0.002) and also, the 2D : 4DL (*P* = 0.005) on fasting plasma HDL-C. The relationship between HDL-C and the 2D : 4D ratio was positive in females but negative in males. The 2D : 4DR accounted for about 54.9% and 46.0% while the 2D : 4DL accounted for about 48.2% and 14.0% of the variabilities in fasting plasma HDL-C in females and males, respectively. Prenatal hormone exposure may partly account for the sexual dimorphism in adult lipid homeostasis.

## 1. Introduction

There is sexual dimorphism in lipid homeostasis as premenopausal women tend to have relatively higher high-density lipoprotein cholesterol (HDL-C), lower atherogenic low-density lipoprotein cholesterol (LDL-C), and triglycerides than similarly-aged males [1]. This occurrence confers protection on females against cardiometabolic disorders before menopause. However, postmenopausal women or women with hyperandrogenaemia due to polycystic ovarian syndrome tend to have poor lipid homeostasis and increased frequency of cardiometabolic diseases. Estrogen has been suggested to possess hypolipidemic properties such that, although LDL-C, very-low-density lipoprotein (VLDL) and triglycerides synthesis may be higher in females than in males, their clearance rate is higher in females due to the estrogen-induced lipoprotein lipase activity [1, 2]. Also, males and females may not differ significantly in their plasma HDL-C, however, females have larger HDL-C than males. The sexual dimorphism in HDL-C concentration has been suggested to be associated with a greater rate of HDL-C apolipoprotein A-I synthesis in women than in men. It has been suggested that sex differences in enzyme expression in lipid homeostasis may be dependent on variation in regulatory proteins which seem to be related to the presence of estrogen, acting through its receptors: Estrogen Receptor beta (ER*β*), Estrogen Receptor alpha (ER*α*), and G-protein coupled Estrogen Receptor (GPER) [1–3]. Although still controversial, estrogen has also been implicated in reverse cholesterol transport (RCT) and cholesterol efflux capacity of HDL-C from macrophages whereby cholesterol from peripheral tissues is transported to the liver to be converted to bile acids and subsequently excreted through the gut [3].

There have been suggestions that prenatal testosterone (PT) and estrogen (PE) exposure may be positively correlated with adult circulating testosterone and estrogen, an indication of a possible association between foetal and adult gonadal activities. However, findings from previous studies have not been consistent [4–6]. Similarly, PT and PE exposures have been associated with sexual dimorphism in the second-to-fourth digit ratio (2D : 4D) such that males tend to have a lower 2D : 4D ratio than females on average. High PT and low PE exposure have been linked to low 2D : 4D ratio and the reverse for increased digit ratio. Asymmetry of the right-left 2D : 4D ratio (Dr-l) has also been demonstrated to be similar in pattern to the 2D : 4D ratio [7]. Although there has not been consensus on the subject, the 2D : 4D ratio and the Dr-l are regarded as the putative markers of prenatal hormone exposure and a cheaper alternative to hormonal assay from amniocentesis [8, 9]. Despite the inconclusive nature of previous studies, some authors sought to determine the association between the 2D : 4D ratio and risk factors for cardiometabolic diseases such as fasting plasma lipid parameters and anthropometric variables. While some authors have reported a positive relationship between the 2D : 4D ratio and the risk of cardiometabolic diseases, others found the reverse or no significant relationship [10–21]. However, a previous study found a significant positive correlation between triglycerides and the 2D : 4D ratio of both hands [22].

The 2D : 4D ratio has been proposed as a tool that may be used for diagnosis, prognosis, and early intervention in lifestyle diseases that may delay the onset of their early detection [23, 24]. If there is a strong relationship between the 2D : 4D ratio and fasting lipid parameters, this will serve as a simpler and cheaper predictive tool for better detection and management of cardiovascular conditions, especially in developing countries such as Ghana. Although some studies on 2D : 4D studies have been conducted in Ghana, none has sought to determine its association with fasting lipids [25, 26]. The study is aimed at determining if there are sex-2D : 4D interactions on fasting lipid variables.

## 2. Materials and Methods

### 2.1. Study Design and Setting

The study was cross-sectional from June to December 2021 at the University for Development Studies (UDS). The university was the first public university to be established in the northern part of Ghana. It started as a single-campus university but has now expanded into a multicampus institution offering both undergraduate and postgraduate programs in Medical, Pharmaceutical, and Nutritional sciences as well as programs in Education and Nursing/Midwifery.

### 2.2. Study Population

The study participants were 206 (female = 93, male = 113) who were between 18 and 30 years. The participants had no known history of fractures that could markedly affect their digit ratio measurement or standing height. They also had no known history of dyslipidaemia or metabolic syndrome. Participation was voluntary and was not restricted by cultural identity, religion, or program of study.

### 2.3. Measurements

#### 2.3.1. Anthropometrics

The standing height (centimeters) and body weight (Kilograms) were measured following recommended guidelines using a stadiometer and bathroom scale, respectively. The palmar surface of the right and left hands was scanned at 150 dpi according to anthropometric standards on the Hp desk jet 2620 all-in-one printer scanner. The lengths of the second and fourth digits were measured to the nearest 0.01 mm using a digital caliper from the hand scans in the GIMP program [(v 2.10.22), http://www.gimp.org]. Each measurement was taken twice, from the computer screen, by one observer and then averaged. The intraclass correlation coefficients (ICC) were calculated by employing the two-way mixed, single measures with an absolute agreement technique. The ICC were 0.950 and 0.966 for the 2D : 4DR and 2D : 4DL, respectively. The Dr-l was derived from the differences between the right and the left 2D : 4D ratios.

#### 2.3.2. Fasting Lipid Variables

The participants were prepared earlier on the requirements for a fasting plasma lipid test based on standard operating procedures [27]. A single venous blood sample was collected by venipuncture after an overnight fast (10-12 hrs). The blood was dispensed into K_2_EDTA and gel separator vacutainer tubes. The blood was stored at 4°C and allowed to clot (gel separator tube). Both tubes were then centrifuged at 3000 rpm for 10 minutes to obtain serum/plasma. The samples were stored at -25°C before analysis without thawing and refreezing. The serum/plasma was then analyzed for total cholesterol (TCHOL), high-density lipoprotein cholesterol (HDL-C), low-density lipoprotein cholesterol (LDL-C), very low-density lipoprotein (VLDL-C), triglycerides (TRIG), and coronary risk (CR) on the BT 1500 automated biochemistry analyzer (Biotecnica Instruments, SPA, Italy) following the manufacturer's instructions and using the recommended reagents.

### 2.4. Statistical Analysis

The data were collected and analyzed in SPSS (v23) and GraphPad Prism (v8). Descriptive statistics were performed for each variable and were presented as the mean ± standard deviation (SD). The mean differences between males and females were tested using Student's *t*-test. Asymmetry in the right-left 2D : 4D ratio (Dr-l) was compared to zero (0) using the one sample, one-tailed *t*-test. To avoid or reduce collinearity between variables, the digit ratio variables were centered on their mean by subtracting the mean value from the variable. Two-way interaction terms were then created by multiplying the centered variable by the sex variable (e.g., Sex∗2D : 4DR-centered). Linear regression models were formulated by entering sex, centered digit ratio, and the interaction term as predictors and a lipid parameter as the dependent variable. To reduce confounding, age and BMI were also entered into the model as covariates. The predicted and residual values of the models with significant main and/or interaction effects were plotted using histograms, probability-probability plots (P-P plots), and scatter plots to test the normality, linearity, and homoscedasticity of the linear regression models, respectively. The unstandardized predicted values of the dependent variable were saved and then plotted on the *Y*-axis against the independent variable (centered on the mean) on the *X*-axis. The sex variable was then used as the marking variable. Statistical significance was determined at *P* < 0.050.

### 2.5. Ethical Declaration

The recommendations of the declaration of Helsinki, 1964 were followed for human subject studies. The study was also approved by the institutional review board of the University for Development Studies. All participants gave written informed consent before the study.

## 3. Results

### 3.1. General Characteristics

The general characteristics of the study population are shown in Table 1. Females were characterized by leftward asymmetry while males showed rightward asymmetry in their right-left 2D : 4D digit ratio (Dr-l). The Dr-l significantly differed from zero, negatively in females (*P* = 0.035) but positively in males (*P* = 0.004). There were no significant differences in the 2D : 4D ratios or lipid parameters between males and females.

### 3.2. The 2D : 4D Ratio and Fasting Lipid Variables

**Figure 1:**
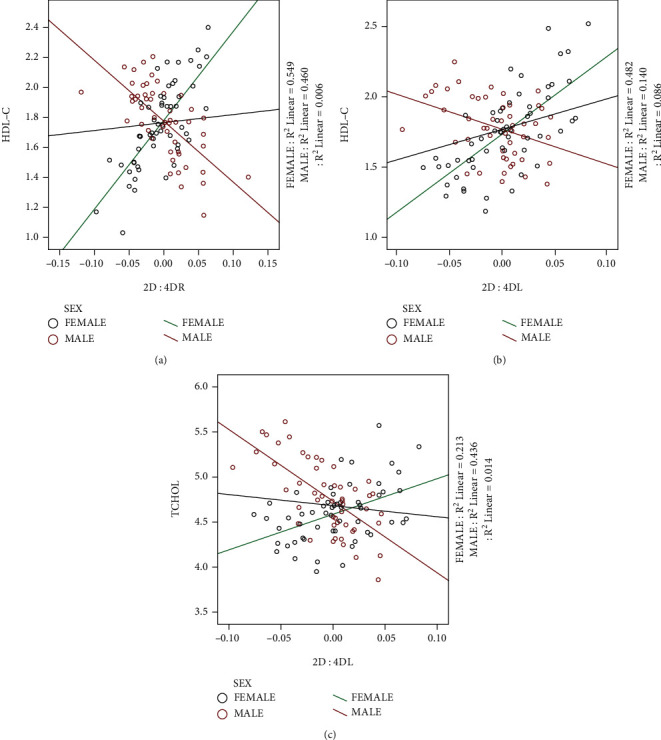
Linear regression plots with interactions between sex and 2D : 4D ratio on lipid variables. Interactions between sex and 2D : 4DR on HDL-C (a); interactions between sex and 2D : 4DL on HDL-C (b); interactions between sex and 2D : 4DL on TCHOL (c). The 2D : 4D values on the *X*-axis were centered on the mean, and the values on the *Y*-axis are the unstandardized predicted values of the dependent variable (HDL-C and TCHOL).

**Figure 2:**
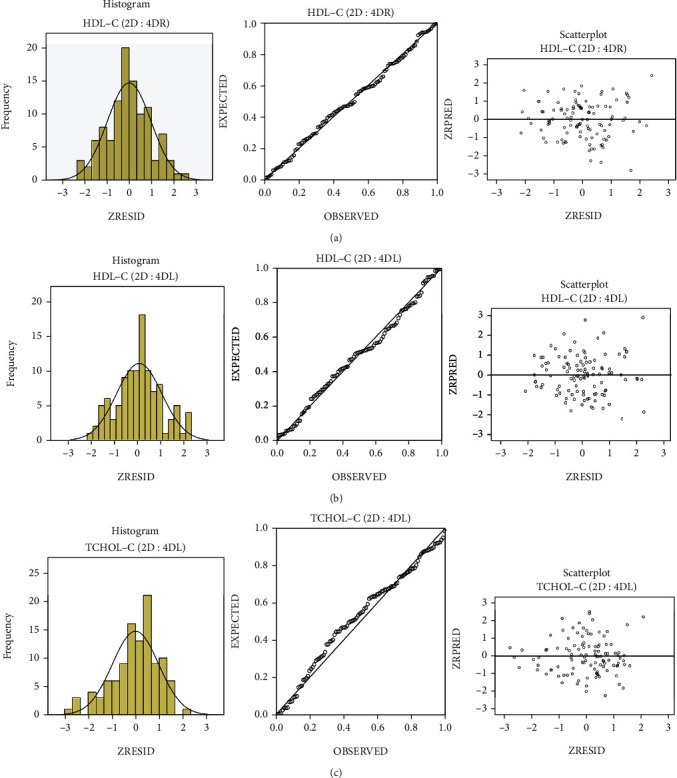
Assumption testing of linear regression. Histogram for normality (column 2), probability-probability plot (P-P plot) for linearity (column 3) and residual versus predicted value scatter plot for homoscedasticity (column 4).

The age and BMI adjusted models fitted with interaction terms are summarized in Tables 2–4 and Figures 1 and 2. The assumptions of the linear regression models were tested using histograms for normality, P-P plots for linearity, and scatter plots for homoscedasticity (Figure 2). The 2D : 4DR had main (*P* = 0.014) and interaction (*P* = 0.002) effects on fasting HDL-C (Table 1). The average adult fasting HDL-C was positively related to the 2D : 4DR which also accounted for about 0.6% of the variability in HDL-C in the total sample (adjR2 = 0.006). However, the 2D : 4DR accounted for 54.9% and 46.0% of the variabilities of HDL-C in adult females and males, respectively. The relationship between the 2D : 4DR and HDL-C was positive in females but negative in males (Figure 1(a)). Similarly, the 2D : 4DL had a main (*P* = 0.007) and interaction (*P* = 0.005) effect on fasting HDL-C (Table 2). The 2D : 4DL accounted for 8.6% of the variability in the average fasting HDL-C with a positive relationship. But in females, the 2D : 4DL accounted for 48.2% of the variability in the fasting HDL-C, while in males it was 14.0%. The relationship was positive in females and negative in males (Figure 1(b)). Also, the 2D : 4DL showed a significant interaction with sex on fasting TCHOL (*P* = 0.008). The relationship was positive in females but negative in males, accounting for 21.3% and 43.6% of the variabilities of TCHOL, respectively (Figure 1(c)).

## 4. Discussion

The study is aimed at determining whether there are interactions between the 2D : 4D ratio and sex on adult fasting lipid variables. The asymmetry in the 2D : 4D ratio was leftward (negative) in females but rightward (positive) in males. The average fasting HDL-C concentration increased with increasing 2D : 4D of both hands. Also, there were significant interactions between sex and the 2D : 4D ratio on HDL-C (both hands) and TCHOL (left hand). The fasting TCHOL and HDL-C concentration increased with an increasing 2D : 4D ratio in females and vice versa in males after controlling for age and body mass index.

Asymmetry of the right-left 2D : 4D ratio (Dr-l) has also been demonstrated to be similar in pattern to the 2D : 4D ratio. Studies have shown that low 2D : 4D on the right hand is associated with more masculine traits while high 2D : 4D on the left hand is related to more feminine traits such that a low Dr-l is a marker of high PT and low PE exposure. It has been explained that the right hand may be more responsive to PT and PE exposure than the left hand [7].

Exposure to high PE and low PT (high 2D : 4D) was significantly associated with increased fasting HDL-C on average which may have also contributed to the positive relationship between the average fasting TCHOL and the 2D : 4D ratio. Some previous studies did not find any significant relationship between the 2D : 4D ratio and HDL-C. But one of the studies was conducted among patients suffering from schizophrenia [28] while the other study involved adolescent girls [29]. Previous studies have suggested that there is a possible correlation between PT and PE exposure and the circulating levels of testosterone and estrogen in adulthood, although findings, including meta-analysis, did not support this observation [5, 6, 30, 31]. In their study, Manning et al. [4] observed that the right and left 2D : 4D ratio of both males and females was positively correlated to their current blood estrogen concentration even after controlling for sex, age, height, and body weight. Also, persons with 2D : 4D > 1.00 were found to have high levels of estrogen compared to those whose 2D : 4D was <1.00. It was also observed that the left 2D : 4D ratio of males was negatively correlated with their current testosterone levels, an indication that foetal and adult testicular activities and the 2D : 4D ratio were positively correlated [4, 6]. But the previous meta-analytic study did not support this finding [5]. One possible reason for the lack of significant association between 2D : 4D and adult circulating testosterone may be that Leydig cells (responsible for testosterone production in males) have different cell populations during the prenatal period, puberty and adulthood. Prenatal Leydig cells degrade after the 24^th^ week of gestation, and the new generation of these cells, which presumably originate from different stem cells, sets on in the postnatal period [32–34].

There is an association between estrogen levels and fasting lipid parameters in adults according to previous studies. Premenopausal women are said to have better lipid homeostasis with a less proatherogenic lipid profile than men although there are ethnic, genetic, and environmental variabilities [35–38]. Although the sex difference is not clearly understood, HDL-C tends to be higher while LDL-C, VLDL-C, and triglycerides levels are lower in premenopausal women than in age-matched men and this has been attributed to the activities of estrogen [2]. Estrogen has been suggested to have hypolipidemic properties whereby the removal of LDL-C particles and VLDL triglycerides from circulation is accelerated through the activation of lipoprotein lipase activity. The sexual dimorphism in HDL-C concentration has been suggested to be associated with a greater rate of HDL-C apolipoprotein A-I synthesis in women than in men [1, 2]. Better lipid homeostasis also means better protection of premenopausal women from hypercholesterolemia and cardiometabolic disease than men. It is suggested that sex differences in enzyme expression may be dependent on variation in regulatory proteins which seem to be related to the presence of estrogen [1, 3]. This suggestion is evidenced in previous studies where an increased 2D : 4D ratio was associated with a reduced risk of metabolic syndrome and/or its complications [20, 21]. However, other studies found higher 2D : 4D in patients with metabolic syndrome and/or its severity, while some found no significant relationships [10–19]. The incidence and the early onset of cardiometabolic diseases have also been found to be relatively higher in males than in premenopausal females. However, these phenomena are reversed in postmenopausal women as estrogen levels begin to decline. This observation has provided pieces of evidence to support the role of estrogen in lipid homeostasis although the process is not that simple due to several confounding factors [2, 36, 39]. Parenteral or oral administration of estrogen has had no or little effect on lipid homeostasis, and in some instances, plasma triglycerides were even elevated [2]. Although the average TCHOL and HDL-C increased with an increasing 2D : 4D ratio, there was sexual dimorphism in the relationship. While the relationship was positive in females, it was rather negative in males. If high PE exposure (high 2D : 4D) correlates positively with adult estrogen levels, then this observation can be explained. Previous studies have indicated that high estrogen in premenopausal women improves insulin sensitivity, while in males it rather increases insulin resistance. Increased insulin resistance is characteristic of metabolic syndrome which may lead to poor lipid homeostasis with low fasting HDL-C [40].

This study is probably the first in Ghana to examine the relationship between the 2D : 4D ratio, a marker of prenatal hormone exposure, and fasting lipids in an adult population. In this study, digit ratios were measured using computer-assisted analysis which is more precise than other techniques [41, 42]. The assumptions of the linear regression models were tested using the residuals and predicted values to ensure goodness-of-fit. The authors, however, acknowledge that there are population variabilities in prenatal hormone exposure and lipid homeostasis [9, 36] and will therefore recommend subpopulation specific studies in Ghana.

## 5. Conclusion

There is a positive relationship between both the right and left 2D : 4D ratio and the average fasting plasma HDL-C. However, there is sexual dimorphism in the effect of prenatal hormone exposure on lipid homeostasis. While the fasting HDL-C increases with increasing 2D : 4D ratio in premenopausal females, the reverse is true for males. Prenatal hormone exposure may partly account for the sexual dimorphism in adult lipid homeostasis. Also, the 2D : 4D ratio may be used as a simple screening tool for lipid homeostasis in adulthood.

## Figures and Tables

**Table 1:** General characteristics of the study population.

Variable	Female *n* = 93	Male *n* = 113	*P* value
BMI (kg/m^2^)	23.6 ± 3.7	21.7 ± 2.6	0.001
TCHOL (mmol/L)	4.67 ± 1.055	4.76 ± 1.080	0.587
TRIG (mmol/L)	1.21 ± 0.558	1.34 ± 0.625	0.209
HDL-C (mmol/L)	1.79 ± 0.685	1.73 ± 0.625	0.595
LDL-C (mmol/L)	2.34 ± 0.827	2.42 ± 0.808	0.556
VLDL-C (mmol/L)	0.55 ± 0.254	0.610.285	0.207
CR	3.91 ± 1.192	4.34 ± 2.600	0.195
2D : 4DR	0.939 ± 0.038	0.946 ± 0.039	0.304
2D : 4DL	0.945 ± 0.038	0.934 ± 0.033	0.071
Dr-l	−0.006 ± 0.027^∗^	0.012 ± 0.035^∗∗^	0.001

Results are presented as the mean ± SD. Male and female variables were compared using unpaired Student's *t*-test (2-tailed). The Dr-l was compared to zero using the one-tailed one sample *t*-test. Significance was measured at ^∗^*P* = 0.035 and^∗∗^*P* = 0.004. TCHOL: total cholesterol; TRIG: triglycerides; HDL: high-density lipoprotein cholesterol; LDL: low-density lipoprotein; VLDL: very low-density lipoprotein; C: cholesterol; CR: coronary risk; BMI: body mass index.

**Table 2:** Interactions between sex and the 2D : 4DR on fasting plasma lipid variables.

LR model	Dependent variable	*B*	95% CI		*P* value
			Lower	Upper	
1	TCHOL (mmol/L)				
	(Constant)	4.932	2.797	7.068	<0.001
	Age (years)	0.061	-0.021	0.143	0.141
	BMI (kg/m^2^)	-0.071	-0.136	-0.006	0.033
	Sex	-0.040	-0.466	0.387	0.854
	2D : 4DR	7.379	-0.530	15.288	0.067
	Sex∗2D : 4DR	-9.392	-20.055	1.270	0.084

2	TRIG (mmol/L)				
	(Constant)	1.001	-0.169	2.172	0.093
	Age	0.012	-0.032	0.057	0.589
	BMI	-0.004	-0.040	0.033	0.836
	Sex	0.092	-0.139	0.324	0.431
	2D : 4DR	1.680	-2.735	6.095	0.452
	Sex∗2D : 4DR	-0.586	-6.469	5.297	0.844

3	HDL-C (mmol/L)				
	(Constant)	2.271	0.946	3.596	0.001
	Age (years)	0.038	-0.013	0.089	0.143
	BMI (kg/m^2^)	-0.057	-0.098	-0.017	0.006
	Sex	-0.136	-0.400	0.129	0.311
	2D : 4DR	6.177	1.269	11.085	0.014
	Sex∗2D : 4DR	-10.480	-17.097	-3.864	0.002

4	LDL-C (mmol/L)				
	(Constant)	2.101	0.412	3.789	0.015
	Age (years)	0.017	-0.048	0.081	0.609
	BMI (kg/m^2^)	-0.006	-0.059	0.047	0.820
	Sex	0.049	-0.286	0.383	0.773
	2D : 4DR	-0.384	-6.755	5.987	0.905
	Sex∗2D : 4DR	2.116	-6.373	10.606	0.622

5	VLDL-C (mmol/L)				
	(Constant)	0.456	-0.076	0.988	0.092
	Age (years)	0.006	-0.015	0.026	0.589
	BMI (kg/m^2^)	-0.002	-0.018	0.015	0.831
	Sex	0.042	-0.063	0.148	0.429
	2D : 4DR	0.793	-1.216	2.801	0.435
	Sex∗2D : 4DR	-0.288	-2.964	2.388	0.831

6	CR				
	(Constant)	1.175	-3.370	5.719	0.609
	Age (years)	0.025	-0.150	0.199	0.780
	BMI (kg/m^2^)	0.095	-0.044	0.233	0.180
	Sex	0.594	-0.313	1.502	0.197
	2D : 4DR	-3.882	-20.711	12.948	0.648
	Sex∗2D : 4DR	17.062	-5.627	39.751	0.139

The 2D : 4DR was centered before linear regression (LR) analysis. TCHOL: total cholesterol; TRIG: triglycerides; HDL: high-density lipoprotein cholesterol; LDL: low-density lipoprotein; VLDL: very low-density lipoprotein; C: cholesterol; CR: coronary risk; BMI: body mass index.

**Table 3:** Interactions between sex and the 2D : 4DL on fasting plasma lipid variables.

LR model	Dependent variable	*B*	95% CI		*P* value
			Lower	Upper	
7	TCHOL (mmol/L)				
	(Constant)	4.533	2.420	6.647	0.000
	Age (years)	0.088	0.005	0.170	0.037
	BMI (kg/m^2^)	-0.082	-0.147	-0.016	0.015
	Sex	-0.097	-0.523	0.329	0.653
	2D : 4DL	4.957	-2.363	12.276	0.182
	Sex∗2D : 4DL	-15.666	-27.192	-4.140	0.008

8	TRIG (mmol/L)				
	(Constant)	0.875	-0.300	2.050	0.143
	Age (years)	0.019	-0.027	0.065	0.413
	BMI (kg/m^2^)	-0.005	-0.042	0.032	0.780
	Sex	0.079	-0.157	0.314	0.509
	2D : 4DL	0.837	-3.235	4.908	0.684
	Sex∗2D : 4DL	-2.897	-9.296	3.502	0.371

9	HDL-C (mmol/L)				
	(Constant)	2.246	0.910	3.583	0.001
	Age (years)	0.045	-0.007	0.097	0.088
	BMI (kg/m^2^)	-0.065	-0.106	-0.024	0.002
	Sex	-0.126	-0.396	0.143	0.354
	2D : 4DL	6.457	1.830	11.085	0.007
	Sex∗2D : 4DL	-10.471	-17.758	-3.184	0.005

10	LDL-C (mmol/L)				
	(Constant)	1.802	0.129	3.475	0.035
	Age (years)	0.032	-0.033	0.097	0.334
	BMI (kg/m^2^)	-0.007	-0.060	0.045	0.783
	Sex	-0.016	-0.351	0.320	0.927
	2D : 4DL	-2.243	-8.039	3.554	0.445
	Sex∗2D : 4DL	-3.441	-12.551	5.669	0.455

11	VLDL (mmol/L)				
	(Constant)	0.397	-0.137	0.932	0.144
	Age (years)	0.009	-0.012	0.029	0.412
	BMI (kg/m^2^)	-0.002	-0.019	0.014	0.777
	Sex	0.036	-0.071	0.143	0.506
	2D : 4DL	0.390	-1.463	2.242	0.677
	Sex∗2D : 4DL	-1.327	-4.238	1.585	0.368

12	CR				
	(Constant)	1.007	-3.595	5.610	0.665
	Age (years)	0.018	-0.161	0.198	0.839
	BMI (kg/m^2^)	0.110	-0.032	0.251	0.129
	Sex	0.620	-0.308	1.547	0.188
	2D : 4DL	-8.417	-24.359	7.524	0.297
	Sex∗2D : 4DL	17.189	-7.913	42.291	0.177

The 2D : 4DL was centered before linear regression (LR) analysis. TCHOL: total cholesterol; TRIG: triglycerides; HDL: high-density lipoprotein cholesterol; LDL: low-density lipoprotein; VLDL: very low-density lipoprotein; C: cholesterol; CR: coronary risk; BMI: body mass index.

**Table 4:** Interactions between sex and the Dr-l on fasting plasma lipid variables.

LR model	Dependent variable	*B*	95% CI		*P* value
			Lower	Upper	
13	TCHOL (mmol/L)				
	(Constant)	4.589	2.467	6.712	0.000
	Age (years)	0.073	-0.010	0.155	0.083
	BMI (kg/m^2^)	-0.068	-0.135	-0.002	0.044
	Sex	-0.062	-0.497	0.373	0.778
	Dr-l	2.839	-7.389	13.068	0.583
	Sex∗Dr-l	3.372	-9.932	16.676	0.616

14	TRIG (mmol/L)				
	(Constant)	0.864	-0.280	2.009	0.137
	Age (years)	0.018	-0.026	0.063	0.417
	BMI (kg/m^2^)	-0.004	-0.040	0.033	0.840
	Sex	0.067	-0.167	0.301	0.570
	Dr-l	1.054	-4.502	6.610	0.707
	Sex∗Dr-l	2.097	-5.088	9.283	0.564

15	HDL-C (mmol/L)				
	(Constant)	2.143	0.778	3.507	0.002
	Age (years)	0.037	-0.016	0.090	0.166
	BMI (kg/m^2^)	-0.053	-0.096	-0.010	0.015
	Sex	-0.065	-0.345	0.215	0.644
	Dr-l	-1.730	-8.307	4.846	0.603
	Sex∗Dr-l	-0.637	-9.191	7.917	0.883

16	LDL-C (mmol/L)				
	(Constant)	1.998	0.378	3.618	0.016
	Age (years)	0.026	-0.037	0.088	0.421
	BMI (kg/m^2^)	-0.009	-0.060	0.042	0.726
	Sex	-0.032	-0.363	0.299	0.850
	Dr-l	3.483	-4.382	11.348	0.382
	Sex∗Dr-l	3.549	-6.623	13.720	0.490

17	VLDL-C (mmol/L)				
	(Constant)	0.392	-0.129	0.913	0.138
	Age (years)	0.008	-0.012	0.028	0.415
	BMI (kg/m^2^)	-0.002	-0.018	0.015	0.839
	Sex	0.031	-0.076	0.137	0.569
	Dr-l	0.507	-2.020	3.035	0.691
	Sex∗Dr-l	0.935	-2.334	4.203	0.572

18	CR				
	(Constant)	0.922	-3.593	5.437	0.686
	Age (years)	0.042	-0.133	0.218	0.633
	BMI (kg/m^2^)	0.092	-0.049	0.233	0.198
	Sex	0.413	-0.513	1.339	0.379
	Dr-l	8.919	-12.839	30.678	0.418
	Sex∗Dr-l	1.111	-27.191	29.412	0.938

The Dr-l was centered before linear regression (LR) analysis. TCHOL: total cholesterol; TRIG: triglycerides; HDL: high-density lipoprotein cholesterol; LDL: low-density lipoprotein; VLDL: very low-density lipoprotein; C: cholesterol; CR: coronary risk; BMI: body mass index.

## Data Availability

The data supporting the findings of this study will be made available upon a reasonable request through the corresponding author.
